# Transcription factor *LHX9* (LIM Homeobox 9) enhances pyruvate kinase PKM2 activity to induce glycolytic metabolic reprogramming in cancer stem cells, promoting gastric cancer progression

**DOI:** 10.1186/s12967-023-04658-7

**Published:** 2023-11-18

**Authors:** Hongying Zhao, Rongke Jiang, Zhijing Feng, Xue Wang, Chunmei Zhang

**Affiliations:** 1 Department of Oncology, Xuzhou City Cancer Hospital, Xuzhou Third People’s Hospital, Jiangsu Province, Xuzhou Hospital Affiliated to Jiangsu University, No. 131, Huancheng Road, Gulou District, Xuzhou, 221000 People’s Republic of China; 2 Jiangsu University, Zhenjiang, 212013 People’s Republic of China

**Keywords:** Gastric cancer, Cancer stem cells, Glycolytic metabolic reprogramming, Transcription factors, *LHX9*, Pyruvate kinase, PKM2, TCGA database, MSigDB database

## Abstract

**Background:**

Glycolytic metabolic reprogramming is a phenomenon in which cells undergo altered metabolic patterns during malignant transformation, mainly involving various aspects of glycolysis, electron transport chain, oxidative phosphorylation, and pentose phosphate pathway. This reprogramming phenomenon can be used as one of the markers of tumorigenesis and development. Pyruvate kinase is the third rate-limiting enzyme in the sugar metabolism process by specifically catalyzing the irreversible conversion of PEP to pyruvate.

**Purpose:**

This study aimed to reveal the critical mediator(s) that regulate glycolytic metabolism reprogramming in gastric cancer and their underlying molecular mechanism and then explore the molecular mechanisms by which *LHX9* may be involved in regulating gastric cancer (GC) progression.

**Methods:**

Firstly, we downloaded the GC and glycolysis-related microarray datasets from TCGA and MSigDB databases and took the intersection to screen out the transcription factor *LHX9* that regulates GC glycolytic metabolic reprogramming. Software packages were used for differential analysis, single gene predictive analysis, and Venn diagram. In addition, an enrichment analysis of the glycolytic pathway was performed. Immunohistochemical staining was performed for *LHX9* and PKM2 protein expression in 90 GC patients, and the association between their expressions was evaluated by Spearman's correlation coefficient method. Three human GC cell lines (AGS, NCI-N87, HGC-27) were selected for in vitro experimental validation. Flow cytometry was utilized to determine the stem cell marker CD44 expression status in GCSCs. A sphere formation assay was performed to evaluate the sphere-forming capabilities of GCSCs. In addition, RT-qPCR and Western blot experiments were employed to investigate the tumor stem cell markers OCT4 and SOX2 expression levels in GCSCs. Furthermore, a lentiviral expression vector was constructed to assess the impact of downregulating *LHX9* or PKM2 on the glycolytic metabolic reprogramming of GCSCs. The proliferation, migration, and invasion of GCSCs were then detected by CCK-8, EdU, and Transwell assays. Subsequently, the mutual binding of *LHX9* and PKM2 was verified using chromatin immunoprecipitation and dual luciferase reporter genes. In vivo experiments were verified by establishing a subcutaneous transplantation tumor model in nude mice, observing the size and volume of tumors in vivo in nude mice, and obtaining fresh tissues for subsequent experiments.

**Results:**

Bioinformatics analysis revealed that *LHX9* might be involved in the occurrence and development of GC through regulating glycolytic metabolism. High *LHX9* expression could be used as a reference marker for prognosis prediction of GC patients. Clinical tissue assays revealed that *LHX9* and PKM2 were highly expressed in GC tissues. Meanwhile, GC tissues also highly expressed glycolysis-associated protein GLUT1 and tumor cell stemness marker CD44. In vitro cellular assays showed that *LHX9* could enhance its activity and induce glycolytic metabolic reprogramming in GCSCs through direct binding to PKM2. In addition, the knockdown of *LHX9* inhibited PKM2 activity and glycolytic metabolic reprogramming and suppressed the proliferation, migration, and invasive ability of GCSCs. In vivo animal experiments further confirmed that the knockdown of *LHX9* could reduce the tumorigenic ability of GCSCs in nude mice by inhibiting PKM2 activity and glycolytic metabolic reprogramming.

**Conclusion:**

The findings suggest that both *LHX9* and PKM2 are highly expressed in GCs, and *LHX9* may induce the reprogramming of glycolytic metabolism through transcriptional activation of PKM2, enhancing the malignant biological properties of GCSCs and ultimately promoting GC progression.

**Supplementary Information:**

The online version contains supplementary material available at 10.1186/s12967-023-04658-7.

## Background

Gastric cancer is a common malignancy with epidemiological characteristics that vary by geographic region [1]. The incidence rate is higher in Asia and lower in Europe and the United States [2]. Meanwhile, the high incidence of gastric cancer is associated with factors such as H. pylori infection, poor nutrition, and dietary habits in humans [3]. In addition, gastric cancer is often asymptomatic in its early stages and difficult to diagnose early, so early prevention and screening are essential [4]. With the continuous development of medical technology, the treatment of gastric cancer has been improved and innovated [5]. Traditional surgery, radiotherapy, and chemotherapy are still the main treatments, but with the development of immunotherapy and targeted therapy, the treatment prospect of gastric cancer has become more promising [6]. Currently, immunotherapies called Pembrolizumab have shown promising efficacy in treating gastric cancer, while targeted therapies such as Trastuzumab have revolutionized the survival of gastric cancer patients [7].

Glycolytic metabolic reprogramming refers to the adjustment of energy supply and growth of tumor cells by adjusting their metabolic patterns during the malignant transformation of tumors [8]. This metabolic reprogramming includes multiple aspects, such as glycolysis, cycloisomerization, and oxidative phosphorylation [9]. These reprogramming alterations provide the energy required by tumor cells and promote the processes of proliferation, differentiation, and infiltration of tumor cells [10]. Cancer stem cells play an essential role in the development and progression of gastric cancer and are closely associated with glycolytic metabolic reprogramming [11]. Glycolytic metabolic reprogramming can promote the self-renewal and proliferation of cancer stem cells while also providing the necessary energy pathway for cancer stem cells to survive in an unsuitable environment [12]. Thus, glycolytic metabolic reprogramming has a critical role in the malignant properties of gastric cancer stem cells [11].

Transcription factor *LHX9* (LIM Homeobox 9) is a nuclear transcription factor associated with various tumors [13]. In human cells, overexpression of *LHX9* occurs in many cell lines and is instead considered a biomarker that predicts poor clinical conditions and prognosis [14]. In gastric cancer cells, high expression of *LHX9* is closely associated with the upregulation of genes related to glycolytic metabolism, the proliferation of gastric cancer stem cells, and poor prognosis [15]. Therefore, *LHX9* is considered a potential marker for pathological features and prognosis of gastric cancer, guiding early detection, diagnosis, and treatment [16]. Pyruvate kinase M2 (PKM2) is essential in reprogramming glycolytic metabolism [17]. In gastric cancer, high expression of PKM2 is often accompanied by tumor invasion and poor prognosis [18]. PKM2 plays an essential role in the pathophysiological features of gastric cancer by participating in glycolytic metabolism to promote tumor stem cell survival and proliferation [19].

The expression pattern and structural characteristics of *LHX9* suggest that it encodes a transcription factor that may be involved in controlling the differentiation of several types of neuronal cells [20]. *LHX9* is essential for the proliferation of germinative ridge cells and the production of testosterone by stromal cells [21]. PKM2 is a critical enzyme in the tumor glucose metabolism pathway, which can accelerate aerobic glycolysis to promote tumor growth and proliferation [22–24]. Additionally, the deletion of the PKM2 gene has been found to increase the extent of neuronal damage and lactate metabolism impairment in the hippocampal region following global cerebral ischemia [25].

This study aimed to reveal how the transcription factor *LHX9* mediates the reprogramming of glycolytic metabolism in gastric cancer stem cells through the regulation of PKM2 activity and to explore its molecular mechanism in the progression of gastric cancer. According to the results, both *LHX9* and PKM2 were highly expressed in gastric cancer tissues, and *LHX9* could directly bind and activate PKM2, which affected the glycolytic metabolism of gastric cancer stem cells. In addition, it was found that high expression of *LHX9* could be used as a reference marker for prognosis prediction of gastric cancer patients. These results reveal the critical role of glycolytic metabolic reprogramming of cancer stem cells in the malignant biological properties of gastric cancer and also provide new ideas for the selection of therapeutic targets and prognostic markers, which are expected to help in the early warning and treatment of gastric cancer.

## Materials and methods

### TCGA (The Cancer Genome Atlas) and MSigDB (Molecular Signatures Database) databases to download GC and glycolysis-related microarray datasets

The gastric cancer RNA-seq datasets, including 375 gastric cancer tissue samples and 32 standard gastric mucosa tissue samples, were downloaded from The Cancer Genome Atlas (TCGA) database (https://portal.gdc.cancer.gov/). Clinical and survival information for the gastric cancer cohort (TCGA-STAD) was obtained from the UCSC Xena database (https://xenabrowser.net/). To identify gene sets related to glycolysis, a search was conducted using the keywords "glycolysis" and species "Homo sapiens" in the Gene Set Enrichment Analysis (GSEA) database of the Molecular Signatures Database (MSigDB) (https://www.gsea-msigdb.org/gsea/msigdb/index.jsp).

### RNA-seq differential analysis to screen for differential genes highly expressed in GC samples

The edgeR package in R software performed a differential analysis of GC-related RNA-seq datasets downloaded from the TCGA database. The FDR method was used for p-value correction, and the screening criteria of |logFC|> 2 and adj. pvalue < 0.05 were used to screen out differentially expressed genes that were highly expressed in GC samples, and the associated heat maps and volcano maps were plotted.

Glycolysis-related genes screened from the MSigDB database were intersected with GC-related differential genes, and Venn diagrams were drawn using the Sento academic website (https://www.xiantao.love/products).

### GSEA enrichment analysis

The GC patient genes downloaded from the TCGA database were analyzed by GSEA software with enrichment of the three gene sets screened for association with glycolysis.

### Single gene expression differential analysis

Six candidate genes (*MET, TKTL1, CLDN9, MIOX, LHX9, CHST4*) were screened after the above steps. A single-gene prognosis analysis was performed for each of the 6 genes. *LHX9* is a common transcription factor LHX family, which can be involved in the progression of osteosarcoma, glioma, and ovarian cancer and is associated with the reprogramming of glycolytic metabolism in glioma and ovarian cancer [13, 15, 26]. Therefore, we selected *LHX9* as the target gene for this study. Then, we extracted the expression matrix of *LHX9* in GC samples and standard samples using the R software "limma" package and "edgeR", performed differential analysis, and plotted the relevant box plots.

### Overall survival (OS) analysis and progression-free interval (PFI) analysis were performed to evaluate the survival outcomes

Using the median expression of *LHX9* as a threshold, gastric cancer patients were divided into high-expression and low-expression groups. OS and PFI analyses were performed using R software's "survival" and "survminer" packages. Kaplan–Meier survival curves were visualized using the "ggplot2" package.

### Receiver operating characteristic (ROC) analysis and precision-recall (PR) analysis

Given the high imbalance of the TCGA dataset, the "pROC" package was used to perform ROC analysis and PR analysis on the data, which were then visualized using ggplot2 to draw diagnostic ROC and PR curves to investigate the diagnostic performance of *LHX9* in different survival times for GC, the "timeROC" package was utilized to generate time-dependent ROC curves.

### Independent prognostic analysis and progression-free survival (PFS) analysis

To further understand whether *LHX9* can be used as an independent, influential factor to predict the prognosis of GC patients, we performed univariate and multifactorial prognostic analyses. The association between gene expression or clinicopathological characteristics and the prognosis of GC patients was analyzed by a one-way Cox regression model with gene expression and clinicopathological characteristics (age, gender, and pathological stage) as predictive variables. Then, all factors with P < 0.05 in the univariate prognostic analysis were put into the multi-factor Cox regression model for independent prognostic analysis.

Using the median of *LHX9* expression as the threshold, GC patients were divided into high and low-expression groups. PFS analysis was performed using the R software "survivor" and "survminer" packages, and survival curves were plotted.

### Sample collection

GC tissues from 90 GC patients (taken from the primary tumor site of GC patients) and their adjacent standard tissue samples (taken from the normal gastric tissues more than 5 cm away from the outer edge of the tumor in the same patients) were collected. The GC patients received no treatment preoperatively. The tissues were divided into two parts. One was immediately frozen in liquid nitrogen, fixed with formaldehyde, and embedded in paraffin. This study was approved by the Ethics Committee of Xuzhou City Cancer Hospital, and informed consent was obtained from all patients and their families in strict compliance with the Declaration of Helsinki (2021-02-056-K01).

### Immunohistochemical staining

The immunohistochemical EnVision staining was performed. The routine paraffin-embedded tissue sections were dewaxed with xylene to water, subjected to antigen retrieval by EDTA, incubated at 3% H2O2 to remove endogenous peroxidase, blocked with goat serum, and probed with the primary antibodies (Additional file 8: Table S1) at 4 °C overnight. After washing twice with PBS, the samples were dropped with secondary antibody and incubated at 37 °C for 30 min. After washing, the samples were developed by DAB, counterstained with hematoxylin, differentiated, dehydrated, cleared, and sealed with neutral gum. The immunohistochemical results were determined by semi-quantitative integration based on the intensity of staining and percentage of positive cells: (1) Scores according to the staining intensity of positive cells: no cheerful coloring as 0, pale yellow as 1, brown as 2, dark brown as 3; (2) Scores according to the percentage of positive cells: ≤ 5% as 0, 6% ~ 25%% is 1, 6% to 25% is 1, 26% ~ 50% is 2, 51% ~ 75% is 3, and > 75% is 4. Multiplication of the two scores was taken as the final result: 4 as positive and < 4 as unfavorable.

### In vitro* culture of human GC cells*

Three human GC cell lines (AGS, NCI-N87, and HGC-27) were purchased from China Microbial Strain Search (Bio-72964, Bio-73110, Bio-73048, respectively.) The NCI-N87 cell line was cultured using RPMI-1640 medium, the AGS cell line was cultured using F12 medium, and the HGC-27 cell lines were cultured using DMEM medium with 10% FBS and 1% double antibodies added to the medium.

### Flow cytometry

CD44 antibody (338,804, BioLegend, CA, USA) was assessed by flow cytometry. GC cell lines were counted after trypsin detachment; the cell number was > 1.0 × 106. After resuspension with 1 ml PBS, cells were centrifuged at 1000 RMP for 5 min. Next, 100 ul PBS was retained, and 5 ul PBS was added at 4 °C. After staining for 30 min, the cells were cleaned with 1 ml PBS and centrifuged for 5 min to remove the supernatant. Subsequently, the samples were added with 300 ul PBS. Flow cytometry determined, sorted, and collected the samples, and data were collated using the FlowJo software (TreeStar, Huizhou, China).

### Spherical experiments to identify GCSCs

The CD44-positive cell population collected by the above steps was washed with PBS and cultured in ultra-low attachment plates containing serum-free medium DMEM/F12 supplemented with B27 (1:50), N2 (1:100), EGF (10 ng/ml), and bFGF (20 ng/ml). The culture medium was changed every 5 days. Cell formation was observed on day 1, day 7, and day 14 of the experiment.

### Transfection of cells with plasmids and transduction of cells with lentivirus

Lentiviral vectors were constructed to knock down *LHX9* or knock down PKM2 for GCSCs, and cells were grouped as follows: sh-NC group, sh-*LHX9*-#1 group, sh-*LHX9*-#2 group, sh-*LHX9*-#3 group, sh-PKM2-#1 group, sh-PKM2-#2 group, and sh-PKM2-#3 group. The lentiviral transfection sequence is shown in Additional file 8: Table S2. The transfection operation was performed according to the lentiviral instructions. After transfection, Western Blot identified the transfection; subsequently, the stably transfected cell lines were screened for subsequent experiments.

For the Resuce experiment, the *LHX9* overexpression plasmid vector was constructed, and the vector was constructed: NCBI to find the CDS sequence of *LHX9* (atgctgaacggtaccactctagaggcagccatgctctttcacgggatctccggaggccacatccaaggcatcatggaggagatggagcgcagatccaagactgaggcccgtctggccaaaggcgcccagctcaacggccgcgacgcgggcatgcccccgctcagcccggagaagcccgccctgtgcgccggctgcgggggcaagatctcggacaggtactatctgctggctgtggacaaacagtggcatctgagatgcctgaagtgctgtgaatgtaagctggccctcgagtccgagctcacctgctttgccaaggacggtagcatttactgcaaggaggattactacagaaggttctctgtgcagagatgtgcccgctgccaccttggcatttccgcctcggagatggtcatgcgcgcccgagactctgtctaccacctgagctgcttcacctgctccacttgcaacaagactctgaccacgggcgaccatttcggcatgaaggacagcctggtgtactgccgcgcccacttcgagaccctcttgcaaggagagtatccaccgcagctgagctacacggagctggcggccaagagcggcggcctggccctgccttacttcaacggtacgggcaccgtgcagaaagggcggccccggaagcggaagagcccagcgctgggagtggacatcgtcaattacaactcaggttgtaatgagaatgaggcagaccacttggaccgggaccagcagccttatccaccctcgcagaagaccaagcgcatgcgaacctctttcaagcatcaccagctccggaccatgaaatcctactttgccatcaaccacaacccggatgccaaggacctcaagcagcttgcccagaaaacaggtctgaccaaaagagttttgcaggtttggttccaaaacgcacgagccaaattcagaaggaaccttttgcggcaggagaatgggggtgttgataaagctgacggcacgtcgcttccggccccgccctcagcagacagcg). The enzyme cleavage site (BstBI/BamHI) was introduced to send a fragment of the CDS region gene for generic biosynthesis (cloned into the plvxpuro vector). Plasmid *LHX9*-plvxpuro was transformed into E. coli DH5α receptor cells were cultured overnight on LB plates containing ampicillin, and the plasmid was extracted and verified by enzymatic digestion.

### RT-qPCR and western blot

The total cellular RNA was extracted with the EZBioscienceRNA kit (EZB-RN4, EZBioscience, USA), with its concentration and purity measured, followed by the reverse transcription or storing at -80℃. The cDNA was synthesized following the reverse transcription kit (A0010CGQ, EZBioscience). RT-qPCR was performed using the RT-qPCR kit (A0012-R2, EZBioscience) according to the manufacturer's instructions. GAPDH served as an internal reference. All primer sequences are shown in Additional file 8: Table S3.

Cells containing PMSF were lysed in RIPA (P0013, Beyotime Biotechnology Co., Shanghai, China) to collect the total protein. The nuclear and cytoplasmic proteins were extracted using the nucleus and cytoplasmic protein utilization kit (P0028, Beyotime), strictly following the instructions. Protein concentration was determined using a BCA kit (P0011/P0012, Beyotime). The protein was subjected to 10–12% SDS-PAGE gel electrophoresis and transferred to a polyvinylidene difluoride membrane (1,620,177, BIO-RAD). After blocking with 5% skim milk or 5% bovine serum albumin, the membrane was incubated with the primary antibodies (Additional file 8: Table S4) at 37 °C overnight. The next day, the protein membrane was washed thrice with 1 × TBST at room temperature for 5 min per time. The protein membrane was incubated with a horseradish peroxidase-conjugated secondary antibody at room temperature for 1 h, followed by visualization using an enhanced chemiluminescence reagent and development on the ImageQuantLAS4000C gel imager (GE, USA). The ratio of the gray value of the target band to that of loading control GAPDH was representative of the relative protein level.

### Determination of lactate content and ATP content in GCSCs

After cells were transfected for 24 h, the lactate content of each group was detected using the Lactate Assay Kit (A019-2-1, Nanjing Jiancheng Bio, China) and the ATP Assay Kit (Item No.: S0027, Beyotime, China) to detect the content of ATP in each group of cells. All experimental operations were performed strictly according to the instructions.

### CCK-8 method and EdU assay to detect the proliferation ability of GCSCs

Cells were treated differently, and CellCountingKit-8 (CCK-8) kit (Item No.: C0038, Beyotime, China) was used for cell proliferation experiments. The experiments were performed according to the kit instructions, and 1 μL of CCK-8 reagent was added to each well at 0 h, 24 h, 48 h, 72 h, and 96 h after cell treatment, mixed and incubated in the cell incubator for 1 h. The absorbance was detected at 450 nm. Each group of experiments was repeated three times.

The plates were seeded with 2X10^6^ units/well in 96-well plates and incubated routinely overnight. Dilute EdU solution using DMEM medium to make EdU (Item No.: C10338 and C10310, RiboBio, Guangzhou, China, https://www.ribobio.com/) working solution concentration of 50 μM, add 100 μLEdU working solution per well and incubate for 2-4 h. Add 100μL of 50μMEdU medium per well. Incubate for 2 h. Discard the medium and wash the cells twice with PBS for 5 min each time. 4% paraformaldehyde was fixed for 15 min; 2 mg/ml glycine solution was washed for 3 min, and then PBS was washed for 3 min. 0.5% Triton-100 was added and incubated for 10 min, and Apollo staining solution was added and incubated for 30 min at room temperature; Hoechst reaction solution, incubate for 30 min at room temperature (avoid light); wash 2 times with PBS avoiding light and seal the film for observation.

### Transwell and scratch assays to detect the migration and invasion ability of GCSCs

Matrigel from BD was used to coat the upper chamber surface of the bottom membrane of the Transwell. The Matrigel was placed at 37 °C for 30 min to polymerize into a gel, and the basement membrane was hydrated before use. The cells of each group were cultured in a serum-free medium for 12 h, harvested, and resuspended with a serum-free medium (1 × 10^5^/ml). The culture containing 10% fetal bovine serum was added to the lower chamber, and 100 ul of cell suspension was taken and added to the Transwell. The cells that had not invaded the surface of the Matrigel membrane were gently removed with cotton swabs after incubation at 37 °C for 24 h, and the cells were fixed with 100% methanol. The cells were fixed and stained with 1% toluidine blue (Sigma). The stained invading cells were counted manually in five randomly selected areas under an inverted light microscope (CarlZeiss, Germany), and each experiment was repeated three times.

Use a marker to mark a horizontal line at 1 cm intervals on the back of the 6-well plate, and make sure that each line passes through the corresponding spike hole. Mark at least 4 lines in each well. About 5 × 10^5^ GCSCs were evenly spaced in the wells, and the fusion rate was observed 12 h after injection. A white tip (10 μL size) was used to scratch the horizontal line perpendicular to the back of the spiked wells 24 h after injection. Cells shed after scoring were rinsed using PBS and repeated three times with the addition of a serum-free medium. After scratching, 6-well plates were placed in a 37 ℃, 5% CO_2_ incubator and sampled at the specified time point 0, 24 h. Cell pictures were taken separately. The cell pictures were statistically analyzed using ImageJ software, and seven horizontal lines were randomly selected to calculate the mean value of intercellular distance.

### Dual luciferase reporter gene assay verifies the interconnection of LHX9 and PKM2

The promoter region of the PKM2 gene was amplified from genomic DNA and subcloned into the pGL4-Basic luciferase reporter vector (Promega, USA). Meanwhile, the mutant expression vector was constructed and subcloned into the pGL4-Basic vector (Promega, USA). The wild-type (WT) and mutant (MT) constructs were confirmed by sequencing. Transcription factor *LHX9* expression plasmids were constructed. GCSCs (2.0 × 10^5^) were made for 24-well plates, and transcription factor *LHX9* expression plasmids and PKM2 reporter gene plasmids were introduced into GCSCs at 50 ng/well using X-tremeGENE 9 (Roche, USA) according to the manufacturer's protocol. 24 h after transfection, cells were collected, lysed with lysis buffer (Promega, USA) for lysis, and luciferase activity was detected using a dual luciferase reporter assay system (Promega, USA). The experiment was repeated three times.

### Chromatin immunoprecipitation assay

The sample tube was added with the *LHX9* antibody (sc-515059, SantaCruzBiotechnology, USA, [1–2 μg/100-500 μg total protein (1 ml cell lysate)]), the negative sample tube was added with the IgG antibody, and the positive sample tube was added with the HistoneH (DlB12) XP@ rabbit monoclonal antibody. The EP tube was sealed with a sealing membrane, placed on a rotary shaker, and mixed at 4 °C overnight. Protein G magnetic beads used for ChIP were added and incubated for 2 h. After rinsing, the chromosomes were eluted from the antibody/protein G magnetic beads and uncross-linked. After DNA purification using the centrifugal column, the ChIP enrichment efficiency was determined by agarose gel electrophoresis.

### *Construction of a subcutaneous transplantation tumor model in nude mice for *in vivo* mechanism validation*

Thirty 4-week-old female BALB/c nude mice were purchased from Xuzhou Medical University Animal Experimental Center (Production License: SCXK (Su) 2020–0011, Jiangsu, China), housed in SPF-grade animal experimentation laboratory, individually caged, with a humidity level of 60%-65% and a temperature of 22–25 °C. The mice were allowed to adapt for one week before the start of the experiment, during which their health status was observed. This experimental procedure and animal usage plan have been approved by the Animal Ethics Committee of our institution (2021-02-056-K01).

For the logarithmic growth phase, single-cell suspensions of sh-*LHX9* and sh-NC GCSCs were prepared after digestion with trypsin and adjusted to a concentration of 1 × 10^6^ cells/mL. A 0.2 mL cell suspension was injected subcutaneously into the proper dorsal tissue of the mice. The diameter of the tumor tissue was measured every 4 days, and its volume was calculated using the formula volume = length × width2/2. After 4 weeks of rearing, the mice were sacrificed, and the tumor tissue was excised, photographed, and the data were recorded. Some tissues were immersed in formalin to produce paraffin blocks, while others were used for detecting the expression of *LHX9* and PKM2 and the activity of glycolytic metabolism reprogramming.

### Statistical methods

The experimental data were statistically analyzed using GraphPad Prism 8.0 software (version 8.0.2, GraphPad Software, USA). Numerical variables with a normal distribution were presented as mean ± standard deviation. Paired t-tests were used for comparing data between cancer tissue and adjacent noncancerous tissue. Independent samples t-tests were used to compare the two groups' other numerical variables. One-way analysis of variance (ANOVA) followed by Tukey's post hoc test was used for comparing multiple independent samples. The Pearson correlation analysis was used to examine the association between variables. Two-way ANOVA or repeated measures ANOVA was used for comparing the data at different time points. All experiments were repeated at least three times. All tests were two-tailed, and the significance level (α) was set at 0.05. A p  < 0.05 was considered statistically significant.

## Results

### LHX9 may be involved in the development and progression of GC through the regulation of glycolytic metabolism

**Fig. 1:**
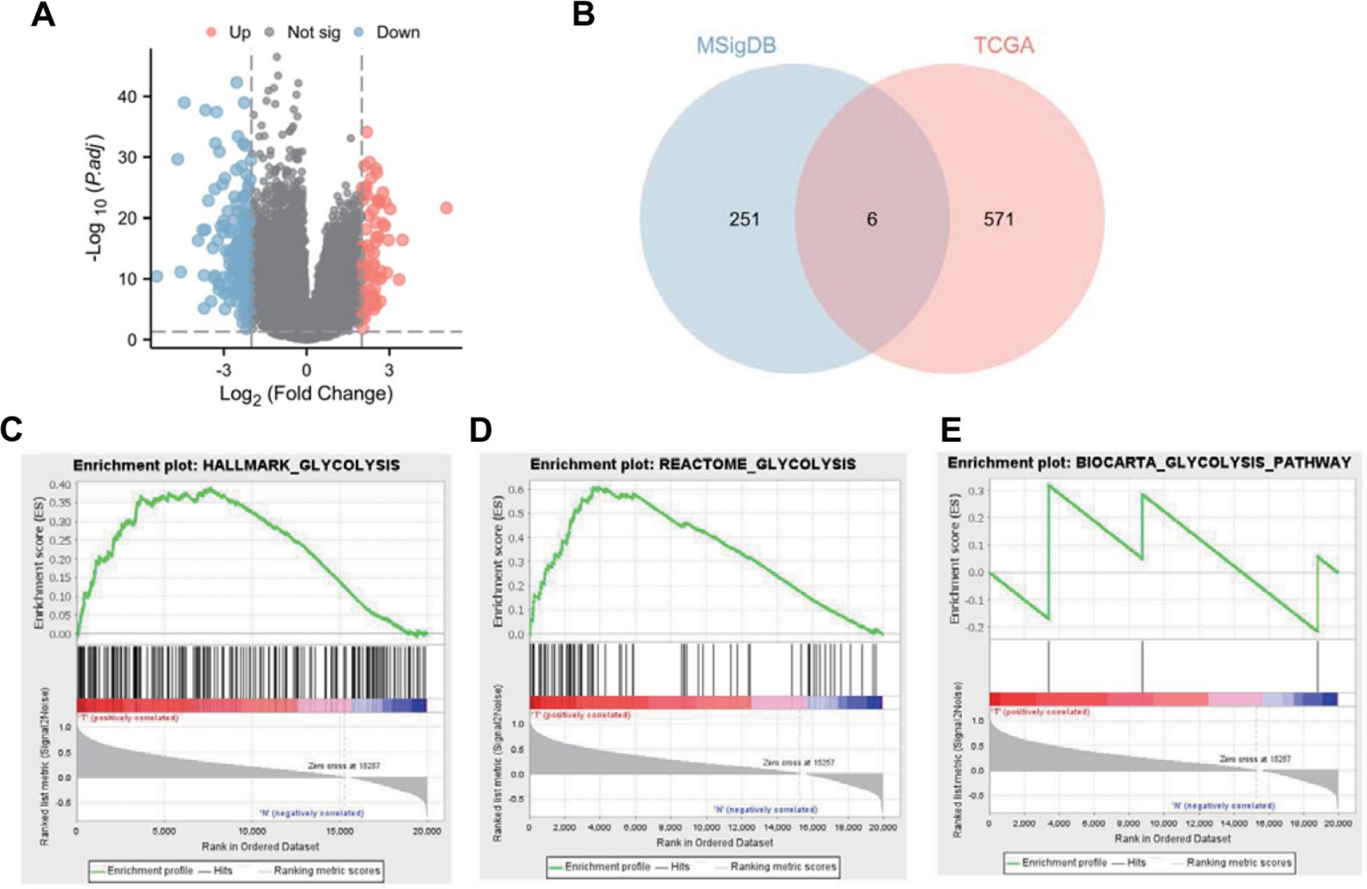
Screening of differential genes associated with GC glycolytic metabolism by combined analysis of TCGA and GSEA databases. **A**: Volcano map of differential genes in GC tissue and normal gastric mucosal tissue, p-value < 0.05 and |logFC|> 2 as the criteria for selection; **B**: Venn diagrams of GC-associated genes intersecting with glycolysis-related genes; **C**: Glycolytic pathway enrichment analysis (HALLMARK_GLYCOLYSIS); **D**: Glycolytic pathway enrichment analysis (REACTOME_ GLYCOLYSIS); **E**: glycolytic pathway enrichment analysis (BIOCARTA_GLYCOLYSIS_PATHWAY)

Firstly, RNA sequencing (RNA-seq) data sets for 375 gastric cancer (GC) tissue samples and 32 standard gastric mucosa tissue samples were downloaded from the TCGA database for differential analysis. Our analysis revealed 577 upregulated genes and 770 down-regulated genes in GC tissue (Additional file 1: Figure S1A, Fig. 1A-B). Subsequently, three gene sets related to glycolysis, comprising 257 genes, were downloaded from the MSigDB database. Next, the intersection between the differentially upregulated genes and the glycolysis-related genes identified six common genes (MET, TKTL1, CLDN9, MIOX, *LHX9*, CHST4) (Fig. 1B). Single-gene survival analysis revealed that CLDN9 and *LHX9* were significantly associated with the prognosis of GC patients (Additional file 8: Table S5). Based on RNA-seq data sets related to GC obtained from the TCGA database, differential expression analysis of MET and TKTL1 in GC tissue and normal gastric mucosa tissue was conducted, showing that MET, TKTL1, CLDN9, MIOX, and CHST4 were highly expressed in GC tissue (Additional file 2: Figure S2-Additional file 6: S6A). GC patients were divided into high-expression and low-expression groups for survival analysis using the median expression level as the cutoff value. The results indicated that the survival prognosis of GC patients in the high- and low-expression groups for MET, TKTL1, MIOX, and CHST4 did not differ significantly, while the low-expression group for CLDN9 had a better survival prognosis than the high-expression group (Additional file 2: Figure S2-Additional file 6: S6B). ROC curve analysis revealed that with an increase in time, the predictive efficiency of MET, CLDN9, MIOX, and CHST4 for the prognosis of GC patients improved, while TKTL1 was not suitable for predicting the survival time of GC patients (Additional file 2: Figure S2-Additional file 6: S6C). Univariate and multivariate survival analyses showed that MET, TKTL1, MIOX, and CHST4 were not independent factors influencing prognosis, whereas CLDN9, patient age, and pathological staging were independent factors affecting prognosis (Additional file 2: Figure S2-Additional file 6: S6D-S6E).

Additionally, the association between the expression levels of each gene and the progression-free interval (PFI) of GC patients was analyzed, indicating that the expression levels of MET, TKTL1, CLDN9, and CHST4 did not affect the PFI of GC patients, while the expression level of MIOX did (Additional file 2: Figure S2F). These findings suggest that high expression of MET, CLDN9, and MIOX can be prognostic markers for GC patients, with better predictive efficiency over time. However, TKTL1 and CHST4 are unsuitable as prognostic markers or independent factors impacting the prognosis of GC patients. Further literature review revealed that *LHX9* is a common transcription factor in the LHX family, which can participate in the progression of osteosarcoma, glioma, and ovarian cancer, and is closely associated with the glycolytic reprogramming of glioma and ovarian cancer [13, 15, 20]. Therefore, *LHX9* was chosen as the target gene for this study. Lastly, gene set enrichment analysis (GSEA) was performed on the three glycolysis pathways involving genes related to GC development, revealing significant enrichment in two pathways (Fig. 1C-E; Additional file 8: Table S6).

### LHX9 and PKM2 were highly expressed in GC tissues

**Fig. 2:**
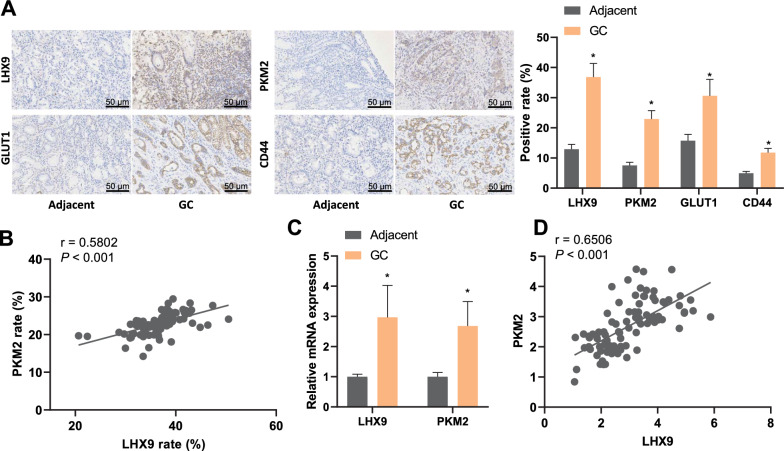
Immunohistochemical detection of protein expression of *LHX9*, PKM2, GLUT1, and CD44 in GC tissues and normal tissues adjacent to cancer. **A**: Immunohistochemical detection of protein expression of *LHX9*, PKM2, GLUT1, and CD44 in GC tissues and normal tissues adjacent to cancer; **B**: Pearson correlation analysis of PKM2 and *LHX9* expression in GC tissues. **C**: The detection of mRNA expression of LHX9 and PKM2 in GC tissues and normal tissues adjacentusing qRT-PCR analysis (N = 90). **D**: Pearson correlation analysis of the relationship between the mRNA expression of LHX9 and PKM2 in GC tissues assessed by qRT-PCR analysis (N = 90). *: P < 0.05, statistically significant comparison between groups. Paired t-tests were conducted to analyze the data between cancer tissue and adjacent tissue, while Pearson correlation analysis was used to measure the relationship between the two variables

Immunohistochemical staining was performed on gastric cancer (GC) and adjacent standard tissue samples. The expression levels of *LHX9* and PKM2 were found to be significantly higher in GC tissue than in normal tissue. Additionally, the glucose metabolism-related protein GLUT1 and the tumor cell stemness marker CD44 were also found to be highly expressed in GC tissue (Fig. 2A). Pearson correlation analysis of the immunohistochemical staining results showed a positive correlation between the expression levels of PKM2 and *LHX9* in GC tissue (Fig. 2B).

qRT-PCR was used to detect the mRNA expression levels of *LHX9* and PKM2 in GC and adjacent normal tissue. The results showed that *LHX9* and PKM2 mRNA were significantly upregulated in GC tissue compared to adjacent tissue (Fig. 2C). Spearman correlation analysis of the qRT-PCR results further confirmed a positive correlation between the expression levels of PKM2 and *LHX9* in GC tissue (Fig. 2D).

These findings suggest that *LHX9* and PKM2 are highly expressed in GC tissue, along with the overexpression of GLUT1 and CD44, indicating their potential involvement in regulating glucose metabolism reprogramming in gastric cancer.

### High LHX9 expression can be used as a reference marker for prognosis prediction in GC patients

**Fig. 3:**
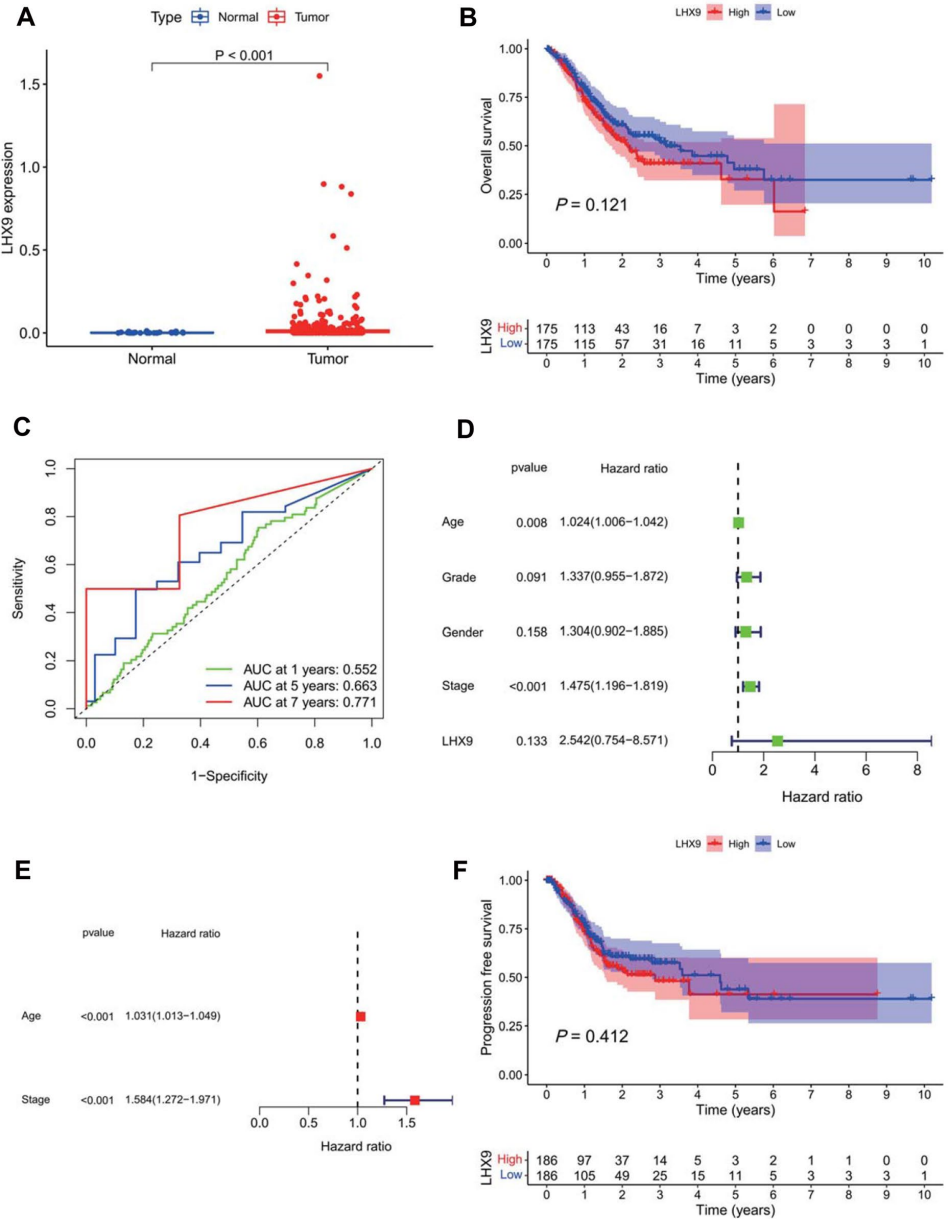
Survival curve and ROC curve to evaluate the predictive value of *LHX9* expression on the prognosis of GC patients. **A**: box line plot of the difference in *LHX9* expression in GC tissues and normal gastric mucosal tissues; **B**: Kaplan–Meier survival analysis curves of GC patients in the high *LHX9* expression group and low *LHX9* expression group; **C-D**: time-dependent ROC curves and and PR curves to evaluate the diagnostic value of *LHX9* on the prognosis of GC patients; **E**: single-factor independent prognostic analysis forest plot; **F**: multi-factor independent prognostic analysis forest plots; G: survival curves to evaluate the predictive efficacy of *LHX9* on PFS in GC patients

Based on the GC-related RNA-seq dataset downloaded from the TCGA database, we further performed a differential analysis of *LHX9* expression in GC tissues and normal gastric mucosal tissues, and the results showed that *LHX9* was highly expressed in GC tissues (Fig. 3A). Using the median of *LHX9* expression as the threshold, GC patients were divided into high and low-expression groups. Survival analysis revealed no significant difference in survival prognosis between GC patients in the *LHX9* high-expression group and *LHX9* low-expression group (Fig. 3B).

ROC curve analysis revealed that the predictive efficacy of *LHX9* on the prognosis of GC patients was higher with increasing time (Fig. 3C). Univariate and multifactorial independent prognostic analyses found that *LHX9* was unavailable as an independent factor affecting prognosis. Still, patient age and pathological stage were available as independent factors of prognosis, and both were high-risk factors for survival in GC patients (Fig. 3D-E). In addition, we also analyzed the association between *LHX9* expression and PFS in GC patients and found that the expression of *LHX9* did not affect the PFS in GC patients (Fig. 3F).

The above results suggest that high *LHX9* expression can be used as a reference marker for prognosis prediction in GC patients, and its predictive efficacy is better with longer survival time, but *LHX9* is not an independent prognostic factor for GC patients.

### The HGC-27 cell line is the most tumor stemness characteristic cell line among the three GC cell lines

It has been reported that GC cells with tumor stemness characteristics of GCSCs express more CD44 protein on their surface and change their cell morphology to a spherical shape after being cultured in special media [27]. Therefore, we screened the cell population with GC stemness by labeling three GC cell lines with CD44 antibodies. Our experimental results revealed that all three GC cell lines could express a large amount of CD44 protein (Additional file 7: Figure S7A). In addition, the results of sphericity experiments showed that: all three GC cell lines could assume a ball shape well after being cultured (Additional file 7: Figure S7B).

These cells were filtered and labeled again with CD44-positive antibodies. Cell populations expressing or not CD44-positive cells were sorted out by flow cytometry and detected by Western Blot and RT-qPCR for the tumor stemness markers OCT4 and SOX2. Our experimental results revealed that the mRNA and protein expression of OCT4 and SOX2 were higher in the CD44-positive cell subpopulations of the three GC cell lines with solid tumor stemness, with the HGC-27 cell line being the highest expressed among the three GC cell lines (Additional file 7: Figure S7C). In addition, Western Blot also confirmed that the HGC-27 cell line was the cell line with the highest expression of *LHX9* and PKM2 proteins among the three (Additional file 7: Figure S7D). Therefore, we concluded that the HGC-27 cell line had the most stemness characteristics and selected the HGC-27 cell line for subsequent experiments.

### Knockdown of LHX9 inhibits PKM2 activity, glycolytic metabolic reprogramming, and malignant biological features of GCSCs

**Fig. 4:**
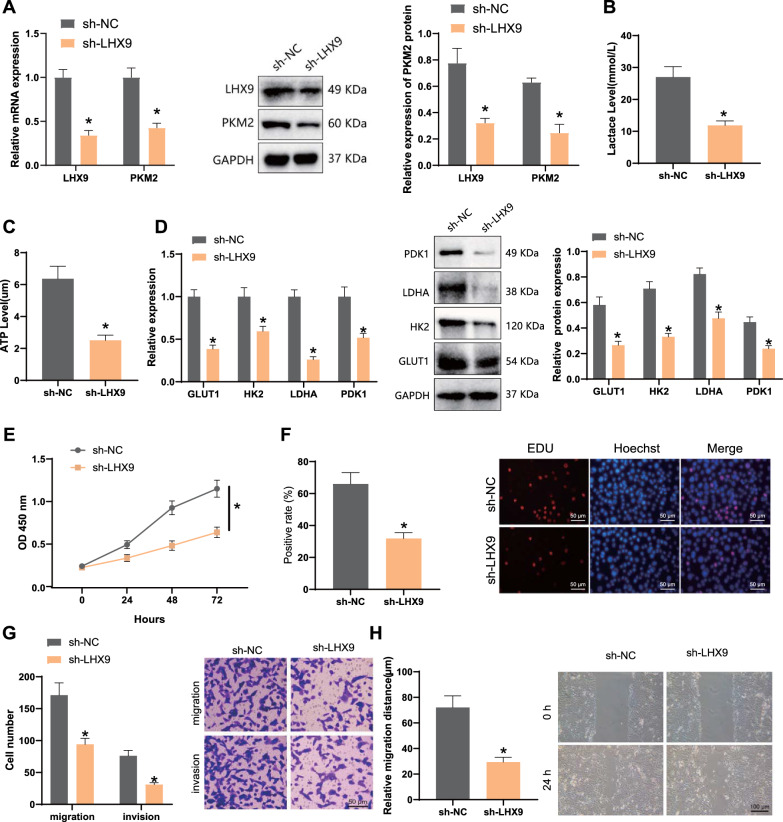
Effect of knockdown of *LHX9* on the reprogramming of glycolytic metabolism and malignant biological properties of GCSCs. **A**: RT-qPCR and Western Blot to detect the effect of knockdown of *LHX9* on PKM2 activity in GCSCs; **B**: changes in lactate levels in GCSCs after knockdown of *LHX9*; **C**: changes in ATP expression in GCSCs after knockdown of *LHX9*; **D**: RT-qPCR and Western Blot to detect the effect of knockdown of *LHX9* on GCSCs transcriptional activity and protein expression changes of glycolysis-related genes (GLUT1, HK2, LDHA, and PDK1) in GCSCs after knockdown of *LHX9*; **E–F**: CCK-8 and EDU assays to detect the effect of knockdown of *LHX9* on the proliferation ability of GCSCs; **G-H**: Transwell and scratch assays to detect the effect of knockdown of *LHX9* on the migration and invasion ability of GCSCs; NC: Human normal gastric epithelial cells (GSE-1); sh-NC: GCSCs transfected with sh-NC by lentivirus; sh-*LHX9*: GCSCs transfected with knockdown *LHX9* by lentivirus; *: P < 0.05, statistically significant comparison between groups; Data comparison between the two groups was conducted using an independent samples t-test. For different time points, a two-factor analysis of variance (ANOVA) was employed to analyze the cellular data. The cell experiments were repeated three times

To investigate the effect of *LHX9* on PKM2 activity, we examined PKM2 expression in GCSCs of the sh-NC group and sh-*LHX9* group by RT-qPCR and Western Blot assay. Our experimental results revealed that both mRNA and protein expression of PKM2 was significantly decreased after knockdown *LHX9* treatment of GCSCs (Fig. 4A).

Then, the effect of *LHX9* on the glycolytic reprogramming of GCSCs was further explored. We compared the lactate level, ATP content, glycolysis-related transcript, and protein expression in GCSCs in the sh-NC and sh-*LHX9* groups. As shown in Fig. 4B, C, the sh-NC group had the highest lactate level and ATP content, and the lactate and ATP levels decreased after knocking down *LHX9*; in addition, the transcriptional activity and protein expression of glycolysis-related genes (GLUT1, HK2, LDHA, and PDK1) in GCSCs were significantly decreased after knocking down *LHX9* (Fig. 4D).

Finally, the effects of *LHX9* on the proliferation, migration, and invasion of GCSCs were examined by CCK-8, EDU, and Transwell assays, which revealed that the knockdown of *LHX9* significantly reduced the proliferation ability of GCSCs (Fig. 4E, F). Similarly, the results of Transwell and scratch experiments revealed that the migration and invasion ability of GCSCs were significantly reduced after knocking down *LHX9* (Fig. 4G, H).

### LHX9 enhances the activity of PKM2 and induces glycolytic reprogramming in GCSCs by directly binding to the promoter region of PKM2. This promotes the malignant biological features of GCSCs

**Fig. 5:**
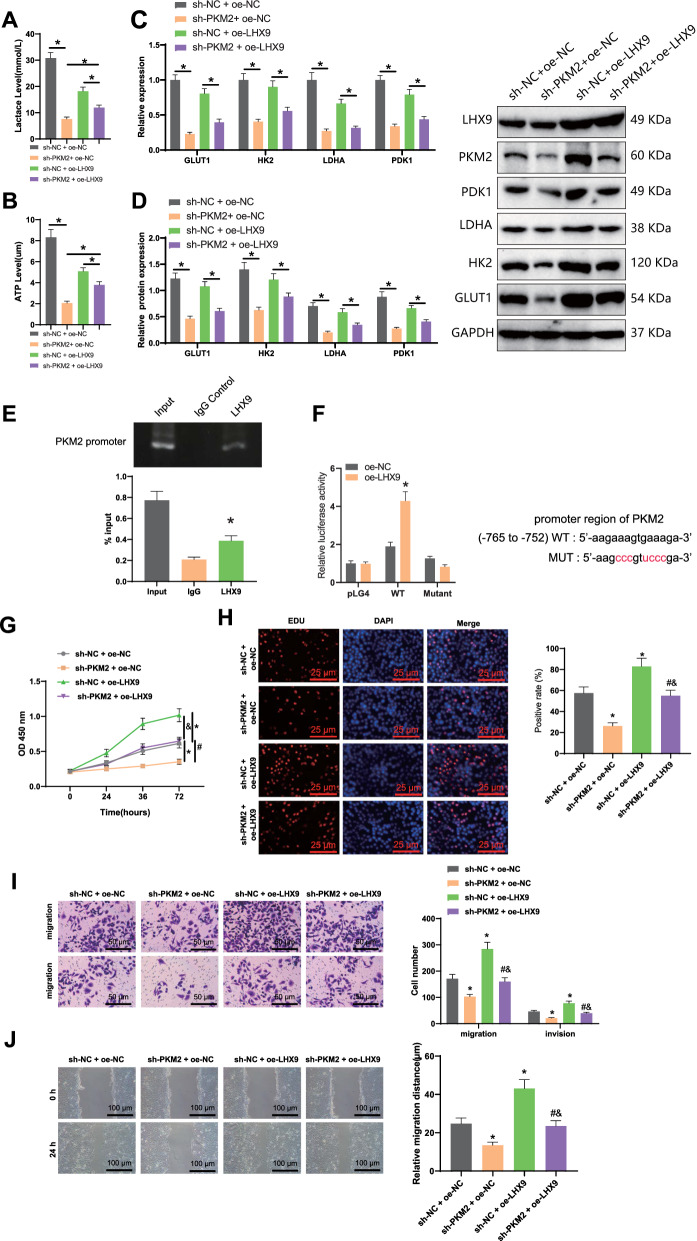
Response experiment to verify the effect of *LHX9*-regulated PKM2 activity on glycolytic metabolic reprogramming in GCSCs. **A**: Effect of knockdown of PKM2 or overexpression of *LHX9* on the expression level of ATP in GCSCs; **B**: Effect of knockdown of PKM2 or overexpression of *LHX9* on the expression level of lactate in GCSCs; **C**: Effect of knockdown of PKM2 or overexpression of *LHX9* on the mRNA and protein expression of glycolysis-related genes (GLUT1, HK2, LDHA, and PDK1) in GCSCs **D**: Effect of knockdown of PKM2 or overexpression of *LHX9* on protein expression of glycolysis-related genes (GLUT1, HK2, LDHA, and PDK1) in GCSCs; E: Chromatin immunoprecipitation assay to verify the interaction of transcription factor *LHX9* and PKM2; **F**: Dual-luciferase reporter gene assay to verify the targeted binding of *LHX9* to PKM2 (transfection of *LHX9* and luciferase activity of PKM2 gene promoter reporter in cells transfected with *LHX9* and empty vector; red area indicates *LHX9* binding site, WT area indicates wild type); **G-H**: The proliferation capacity of GCSCs was evaluated using CCK-8 and EDU methods; **I-J**: The migration and invasion abilities of GCSCs were assessed using Transwell and scratch experiments. *: P < 0.05, compared with the sh-NC + oe-NC group, the IgG group, or the oe-NC group, there was a significant difference; #: P < 0.05, compared with the sh-PKM2 + oe-NC group, there was a significant difference; &: P < 0.05, compared with the sh-NC + oe-*LHX9* group, there was a significant difference. Multiple group comparisons were conducted using one-way analysis of variance (ANOVA), and the cell experiments were repeated three times

We further investigated the impact of *LHX9* regulation on PKM2 activity and the reprogramming of glycolytic metabolism in GCSCs. Firstly, we examined the changes in lactate and ATP levels in each group of GCSCs. As shown in Fig. 5A, B, compared to the sh-NC + oe-NC group, the sh-PKM2 + oe-NC group exhibited a significant decrease in lactate and ATP levels, while the sh-NC + oe-*LHX9* group showed a significant increase in lactate and ATP levels. Furthermore, the sh-PKM2 + oe-*LHX9* group demonstrated a partial recovery in lactate and ATP levels compared to the sh-PKM2 + oe-NC group and an apparent decrease in lactate and ATP levels compared to the sh-NC + oe-*LHX9* group.

Next, we used RT-qPCR and Western blot analysis to assess the mRNA and protein expression of *LHX9*, PKM2, and glycolytic-related genes (GLUT1, HK2, LDHA, and PDK1). The results revealed that, compared to the sh-NC + oe-NC group, the sh-PKM2 + oe-NC group showed no significant difference in *LHX9* mRNA and protein expression. However, it exhibited a significant decrease in the mRNA and protein expression of PKM2 and glycolytic-related genes. On the other hand, the sh-NC + oe-*LHX9* group demonstrated a significant increase in mRNA and protein expression of *LHX9*, PKM2, and glycolytic-related genes. Moreover, compared to the sh-PKM2 + oe-NC group, the sh-PKM2 + oe-*LHX9* group exhibited a significant increase in the mRNA and protein expression of *LHX9*, PKM2, and glycolytic-related genes. Lastly, compared to the sh-NC + oe-*LHX9* group, the sh-PKM2 + oe-*LHX9* group showed no significant difference in *LHX9* mRNA and protein expression but a decrease in the mRNA and protein expression of PKM2 and glycolytic-related genes (Fig. 5C-D).

We conducted chromatin immunoprecipitation and dual-luciferase reporter experiments to investigate the potential direct interaction between *LHX9* and PKM2. Our findings confirmed that *LHX9* can directly bind with PKM2 (Fig. 5E, F).

The involvement of LHX9 in regulating the proliferation, migration, and invasion of GCSCs was assessed through CCK-8, EDU, and Transwell experiments. CCK-8 and EDU experiments revealed that compared to the sh-NC + oe-NC group, the sh-PKM2 + oe-NC group exhibited significantly weakened proliferation, migration, and invasion capabilities of GCSCs. Additionally, the sh-NC + oe-LHX9 group showed significantly enhanced proliferation, migration, and invasion abilities. Comparative analysis with the sh-PKM2 + oe-NC group demonstrated that the sh-PKM2 + oe-LHX9 group had significantly increased proliferation, migration, and invasion capacities (Fig. 5G–J).

These findings suggest that LHX9 enhances PKM2 activity by directly binding to the promoter region of PKM2, leading to the induction of glycolytic metabolic reprogramming in GCSCs, thereby promoting their malignant biological characteristics.

### Knockdown of LHX9 inhibits PKM2 activity and reprograms glycolytic metabolism, thereby reducing the tumorigenic capacity of GCSCs in nude mice

**Fig. 6:**
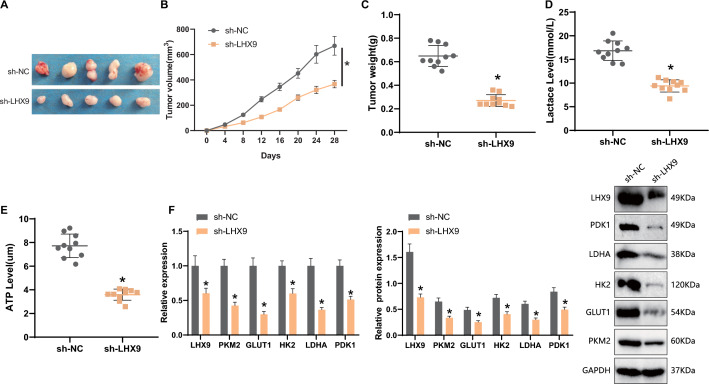
Nude Mouse Subcutaneous Tumor Model to Validate the Effect and Mechanism of *LHX9* on Tumor Growth and Glycolytic Reprogramming. **A**: comparison of subcutaneous transplantation tumor mass in each group of nude mice; **B**: comparison of subcutaneous transplantation tumor volume in each group of nude mice; **C**: comparison of subcutaneous transplantation tumor mass in each group of nude mice; **D**: comparison of lactate expression in subcutaneous transplantation tumor tissue in each group of nude mice; **E**: comparison of ATP content in subcutaneous transplantation tumor tissue in each group of nude mice; **F**: comparison of glycolysis-related genes (GLUT1, HK2, LDHA F: Comparison of mRNA and protein expression of glycolysis-related genes (GLUT1, HK2, LDHA, and PDK1) in subcutaneous transplanted tumor tissues of nude mice in each group; *: P < 0.05, statistically significant between groups; An independent samples t-test was used to compare the two groups, and a repeated ANOVA was employed to analyze the tumor data at different time points (N = 10)

The effect of the *LHX9*/PKM2 axis on the tumorigenic ability of GCSCs in nude mice was observed by constructing a subcutaneous transplantation tumor model. Our experimental results showed that the tumor weight and volume were significantly reduced in the sh-*LHX9* group compared with the sh-NC group (Fig. 6A–C). RT-qPCR and Western Blot were performed on fresh tissues from subcutaneous transplanted tumors of nude mice. The results showed that compared to the sh-NC group, the lactate and ATP contents in tumor tissues were significantly reduced in the sh-*LHX9* group (Fig. 6D, E), and the mRNA and protein expression of glycolysis-related genes (GLUT1, HK2, LDHA, and PDK1) were also significantly reduced (Fig. 6F).

## Discussion

Gastric cancer is a highly dangerous malignancy with high morbidity and mortality [28]. Its occurrence and progression involve multiple factors, including environmental and genetic [29]. In recent years, studies have shown that glycolytic metabolic reprogramming plays an important role in various tumors [30]. PKM2 is a critical enzyme in the glycolytic metabolic pathway, and it has been shown that its expression is increased in a variety of tumors and is associated with cancer stem cells and glycolytic metabolic reprogramming [31]. The transcription factor *LHX9* is a newly identified important regulator that plays a vital role in various physiological and pathological processes [13]. Therefore, an in-depth investigation of the regulatory mechanism of glycolytic metabolic reprogramming by *LHX9* and PKM2 in gastric cancer is expected to provide new strategies and potential targets for treating gastric cancer.

MET is a receptor tyrosine kinase closely associated with cell proliferation, migration, and invasion processes. Studies have shown higher expression levels of MET in GC tissue [32]. This may be related to enhanced proliferation and invasion of GC cells. However, in this study, no significant differences were observed in MET expression levels and the survival prognosis of GC patients, suggesting that MET may not be an independent factor affecting patient survival. TKTL1 is an enzyme-linked to abnormal glucose metabolism and tumor development. However, this study found that TKTL1 expression levels could not be used to predict the survival time of GC patients [33]. This suggests a limited role of TKTL1 in the survival prognosis of GC patients, warranting further research to confirm its specific function and mechanism. CLDN9 is a cell adhesion protein that maintains cell adhesion and tissue structure. This study revealed a significant correlation between low CLDN9 expression in GC tissue and improved survival prognosis [34]. This implies a potential inhibitory role of CLDN9 in the development and prognosis of GC.

Further research aimed at uncovering the precise mechanisms and functions of CLDN9 in GC will contribute to a better understanding of its development and prognosis. In addition, the high expression of MET and CLDN9 may potentially predict the prognosis of gastric cancer patients, whereas the role of TKTL1 remains unclear. These findings offer valuable insights into the prognosis prediction and treatment of gastric cancer. However, further research is needed to validate these results and investigate these molecules' precise mechanisms and roles in the development and prognosis of gastric cancer.

Our study validated that *LHX9* is highly expressed in GC tissues and is involved in GC development by regulating glycolytic metabolism. Specifically, high *LHX9* expression was more significant in predicting effectiveness and prolonged survival as a prognostic marker in GC patients [35]. *LHX9* is a member of the joint LHX transcription factor family and can be involved in the metabolic reprogramming of glioma and ovarian glycolytic metabolism to influence tumor progression [13]. Metabolic pathway intermediates are crucial in regulating GCSCs to influence GC progression [36]. Notably, cellular metabolic reprogramming and glycolysis have been widely recognized in GC [37]. The current study also demonstrated that the downregulation of *LHX9* could inhibit glycolytic metabolic reprogramming and malignant biological features of GCSCs by reducing PDK1, LDHA, GLUT1, and HK2 levels, thereby delaying GC progression [38]. LDHA, GLUT1, and HK2 are known to be glycolysis-promoting enzymes that induce glycolysis [17]. PDK1 is a crucial glycolytic enzyme closely associated with cancer cell proliferation, metastasis, and chemoresistance [39]. It has been reported that elevated PDK1 may lead to increased lactate content, glucose uptake, and ATP production [40]. Many studies have also shown that metabolic reprogramming is critical for cancer cell proliferation, invasion, and metastasis [41]. Another study showed that inhibition of the tumor glycolytic pathway helps to inhibit GC cell proliferation and thus can kill GC cells [42]. High expression of *LHX9* was associated with migration and invasion of osteosarcoma cells [26]. Overexpressed *LHX9* is involved in glycolysis during carcinogenesis, progression, proliferation, and invasion [43]. Therefore, these findings support that *LHX9* knockdown can inhibit the glycolytic metabolic reprogramming and malignant biological features of GCSCs, thereby suppressing GC progression [38].

In addition, PKM2 was shown to be highly expressed in GC tissues. Similarly, recent studies have confirmed that PKM2 is up-regulated in GC cells [44]. *LHX9* deficiency inhibits PKM2 activity and suppresses the reprogramming of glycolytic metabolism to prevent GC progression [45]. Dysregulation of PKM2 is one of the most common pathogenic subtypes in human cancers [45]. The induction of enzymatic activity of PKM2 enhances glycolysis and malignancy in GC [46]. Another study also elucidated that the downregulation of PKM2 inhibited the proliferation and invasive phenotype of GC cells [18]. Further analysis showed that silencing of *LHX9* inhibited the metabolic reprogramming of glycolysis and the malignant biology of GCSCs by reducing PKM2 activity.

## Conclusion

**Fig. 7:**
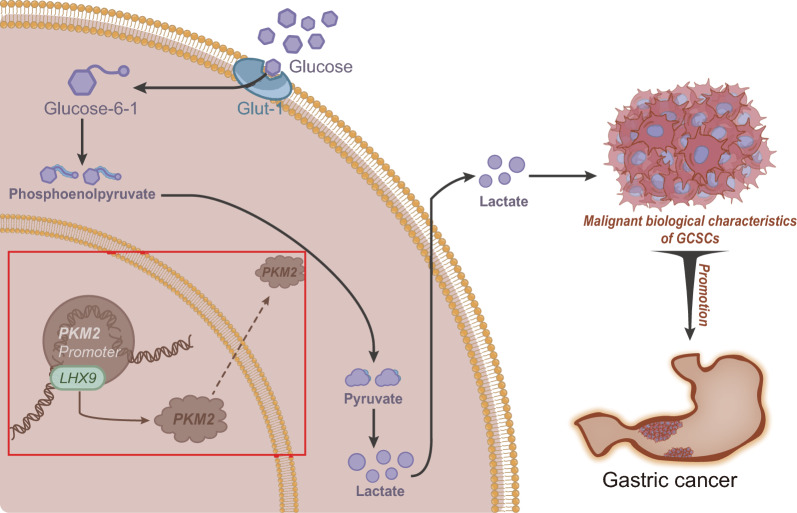
Schematic diagram of the molecular mechanism by which transcription factors *LHX9* regulates pyruvate kinase PKM2 activity to mediate the reprogramming of glycolytic metabolism in cancer stem cells to affect gastric cancer progression. High expression of CLDN9 can be used as an independent influence factor for prognosis prediction in GC patients, and its predictive efficacy is better with longer survival time; high expression of MET and MIOX can be used as reference markers for prognosis prediction in GC patients, and their predictive efficacy is better with longer survival time, but they are not independent prognostic factors for GC patients; high expression of TKTL1 and CHST4 cannot be used as prognostic TKTL1 and CHST4 high expression cannot be used as reference markers and independent impact factors for prognosis of GC patients

In summary, we can tentatively conclude that both *LHX9* and PKM2 are highly expressed in GC, and *LHX9* may induce glycolytic metabolic reprogramming through transcriptional activation of PKM2, enhance the malignant biological properties of GCSCs, and ultimately promote GC progression (Fig. 7). In addition, the knockdown of *LHX9* may be a novel strategy for treating gastric cancer, and this approach may be more effective than existing therapeutic approaches. The limitations of this study are that the study was based on small sample case studies and cellular experiments. Further, extensive sample studies and validation of confirmed cases are needed to explore the molecular mechanism of *LHX9* regulation of gastric cancer progression. In addition, this study only focused on the role of transcription factor *LHX9* and pyruvate kinase PKM2 while ignoring other factors that may be involved in gastric cancer progression.

### Supplementary Information

**Additional file 1: Figure S1.** Heat map of differential genes in GC tissue and normal gastric mucosal tissue.

**Additional file 2: Figure S2**. Survival curve and ROC curve to evaluate the predictive value of MET expression on the prognosis of GC patients. A: box line plot of the difference in MET9 expression in GC tissues and normal gastric mucosal tissues; B: Kaplan–Meier survival analysis curves of GC patients in the high MET expression group and low MET expression group; C: time-dependent ROC curve to evaluate the predictive efficacy of MET on the prognosis of GC patients; D: forest plot of single-factor independent prognostic analysis; E: forest plot of multi-factor independent prognostic analysis; F: survival curves to evaluate the predictive efficacy of MET on PFS in GC patients.

**Additional file 3: Figure S3**. Survival curve and ROC curve to evaluate the predictive value of TKTL1 expression on the prognosis of GC patients. A: box line plot of the difference in TKTL1 expression in GC tissues and normal gastric mucosal tissues; B: Kaplan–Meier survival analysis curves of GC patients in the TKTL1 high expression group and TKTL1 low expression group; C: time-dependent ROC curves to evaluate the predictive efficacy of TKTL1 on the prognosis of GC patients; D: single-factor independent prognostic analysis forest plot; E: multi-factor independent prognostic analysis forest plot; F: survival curves to evaluate the predictive efficacy of TKTL1 on PFS in GC patients.

**Additional file 4: Figure S4.** Survival curve and ROC curve to evaluate the predictive value of CLDN9 expression on the prognosis of GC patients. A: box line plot of the difference in CLDN9 expression in GC tissues and normal gastric mucosal tissues; B: Kaplan–Meier survival analysis curves of GC patients in the high CLDN9 expression group and low CLDN9 expression group; C: time-dependent ROC curves to evaluate the predictive efficacy of CLDN9 on the prognosis of GC patients; D: single-factor independent prognostic analysis forest plot; E: multi-factor independent prognostic analysis forest plot; F: survival curves to evaluate the predictive efficacy of CLDN9 on PFS in GC patients.

**Additional file 5: Figure S5.** Survival curve and ROC curve to evaluate the predictive value of MIOX expression on the prognosis of GC patients. A: box line plot of the difference in MIOX expression in GC tissues and normal gastric mucosal tissues; B: Kaplan–Meier survival analysis curves of GC patients in the high MIOX expression group and low MIOX expression group; C: time-dependent ROC curves to evaluate the predictive efficacy of MIOX on the prognosis of GC patients; D: single-factor independent prognostic analysis forest plot; E: multi-factor independent prognostic analysis forest plots; F: survival curves to evaluate the predictive efficacy of MIOX on PFS in GC patients.

**Additional file 6: Figure S6.** Survival curve and ROC curve to evaluate the predictive value of CHST4 expression on the prognosis of GC patients. A: box line plot of the difference in CHST4 expression in GC tissues and normal gastric mucosal tissues; B: Kaplan–Meier survival analysis curves of GC patients in the high CHST4 expression group and low CHST4 expression group; C: time-dependent ROC curves to evaluate the predictive efficacy of CHST4 on the prognosis of GC patients; D: single-factor independent prognostic analysis forest plot; E: multi-factor independent prognostic analysis forest plot; F: survival curves to evaluate the predictive efficacy of CHST4 on PFS in GC patients.

**Additional file 7: Figure S7.** Screening and identification of GCSCs by flow cytometry, spherical assay, RT-qPCR, and Western Blot. A: flow cytometry to detect the expression of stemness marker CD44 in three GC cell lines (AGS, NCI-N87, HGC-27); B: a spheroid assay to detect spheroid formation on day 1, day 7, and day 14 in three GC cell lines (AGS, NCI-N87, HGC-27); C: RT-qPCR and Western Blot assay to detect the expression of tumor stemness markers OCT4 and SOX2 in the three GC cell lines (AGS, NCI-N87, HGC-27); D: Western Blot assay to detect the expression of *LHX9* and PKM2 in the three GC cell lines (AGS, NCI-N87, HGC-27); *: P < 0.05, statistically significant for comparison between groups significance; The data comparison between the two groups was conducted using an independent samples t-test, and the cell experiments were repeated three times.

**Additional file 8: Table S1.** Source information of the primary antibodies. **Table S2.** Lentivirus transduction sequences. **Table S3.** RT-qPCR primer sequences. **Table S4.** Source information of the primary and secondary antibodies**. Table S5.** Single gene prognostic analysis among six common genes. **Table S6.** Gene Set Enrichment Analysis (GSEA).

## Data Availability

The datasets generated and analyzed during the current study are available from the corresponding author upon reasonable request.

## Abbreviations

GCSCs: Gastric cancer stem cells
GC: Gastric cancer
PKM2: Pyruvate kinase
PFS: Progression-free survival
CCK-8: CellCountingKit-8
WT: Wild-type
MT: Mutant
