# Supplementary figures and images for: Transcription factor LHX9 (LIM Homeobox 9) enhances pyruvate kinase PKM2 activity to induce glycolytic metabolic reprogramming in cancer stem cells, promoting gastric cancer progression
Source: J Transl Med. 2023 Nov 18;21:833. doi: 10.1186/s12967-023-04658-7 (PMC10657563; doi:10.1186/s12967-023-04658-7)

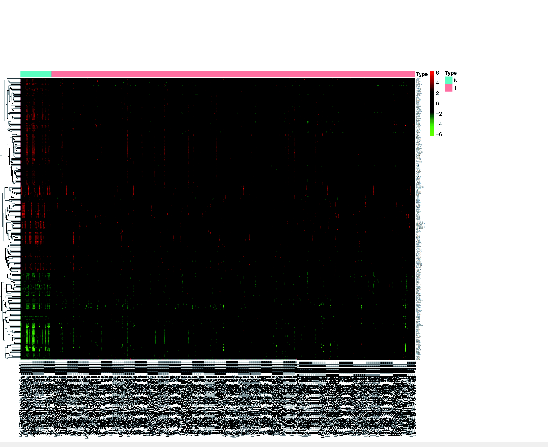

Supplement: Supplementary file 1 — Additional file 1: Figure S1. Heat map of differential genes in GC tissue and normal gastric mucosal tissue. [file 12967_2023_4658_MOESM1_ESM.docx]

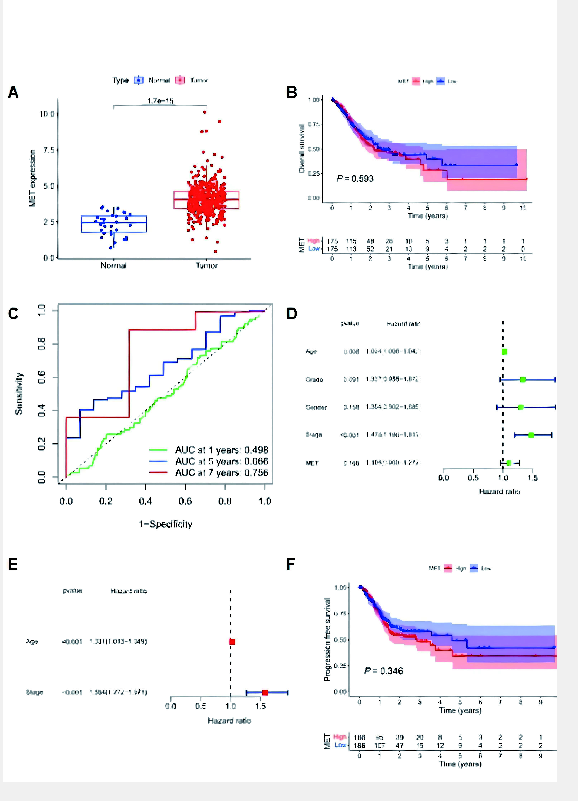

Supplement: Supplementary file 2 — Additional file 2: Figure S2. Survival curve and ROC curve to evaluate the predictive value of MET expression on the prognosis of GC patients. A: box line plot of the difference in MET9 expression in GC tissues and normal gastric mucosal tissues; B: Kaplan–Meier survival analysis curves of GC patients in the high MET expression group and low MET expression group; C: time-dependent ROC curve to evaluate the predictive efficacy of MET on the prognosis of GC patients; D: forest plot of single-factor independent prognostic analysis; E: forest plot of multi-factor independent prognostic analysis; F: survival curves to evaluate the predictive efficacy of MET on PFS in GC patients. [file 12967_2023_4658_MOESM2_ESM.docx]

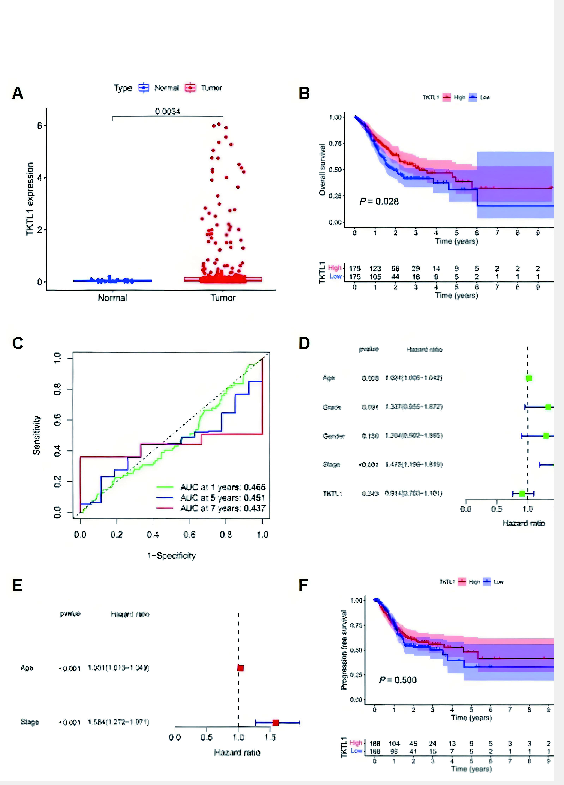

Supplement: Supplementary file 3 — Additional file 3: Figure S3. Survival curve and ROC curve to evaluate the predictive value of TKTL1 expression on the prognosis of GC patients. A: box line plot of the difference in TKTL1 expression in GC tissues and normal gastric mucosal tissues; B: Kaplan–Meier survival analysis curves of GC patients in the TKTL1 high expression group and TKTL1 low expression group; C: time-dependent ROC curves to evaluate the predictive efficacy of TKTL1 on the prognosis of GC patients; D: single-factor independent prognostic analysis forest plot; E: multi-factor independent prognostic analysis forest plot; F: survival curves to evaluate the predictive efficacy of TKTL1 on PFS in GC patients. [file 12967_2023_4658_MOESM3_ESM.docx]

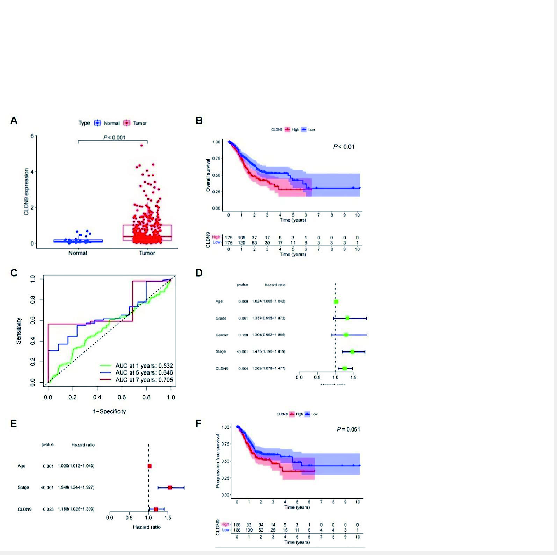

Supplement: Supplementary file 4 — Additional file 4: Figure S4. Survival curve and ROC curve to evaluate the predictive value of CLDN9 expression on the prognosis of GC patients. A: box line plot of the difference in CLDN9 expression in GC tissues and normal gastric mucosal tissues; B: Kaplan–Meier survival analysis curves of GC patients in the high CLDN9 expression group and low CLDN9 expression group; C: time-dependent ROC curves to evaluate the predictive efficacy of CLDN9 on the prognosis of GC patients; D: single-factor independent prognostic analysis forest plot; E: multi-factor independent prognostic analysis forest plot; F: survival curves to evaluate the predictive efficacy of CLDN9 on PFS in GC patients. [file 12967_2023_4658_MOESM4_ESM.docx]

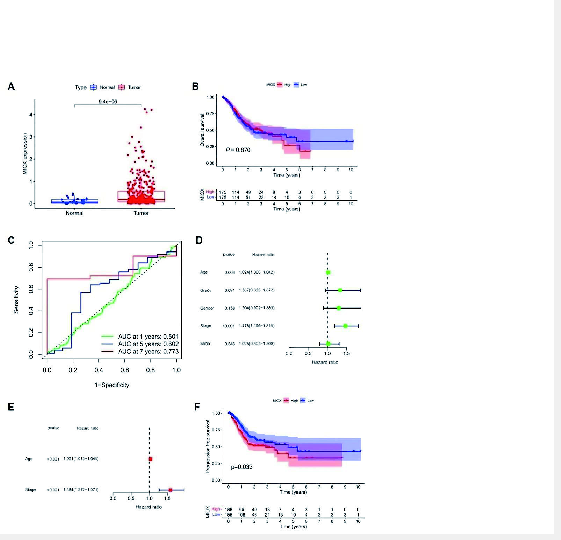

Supplement: Supplementary file 5 — Additional file 5: Figure S5. Survival curve and ROC curve to evaluate the predictive value of MIOX expression on the prognosis of GC patients. A: box line plot of the difference in MIOX expression in GC tissues and normal gastric mucosal tissues; B: Kaplan–Meier survival analysis curves of GC patients in the high MIOX expression group and low MIOX expression group; C: time-dependent ROC curves to evaluate the predictive efficacy of MIOX on the prognosis of GC patients; D: single-factor independent prognostic analysis forest plot; E: multi-factor independent prognostic analysis forest plots; F: survival curves to evaluate the predictive efficacy of MIOX on PFS in GC patients. [file 12967_2023_4658_MOESM5_ESM.docx]

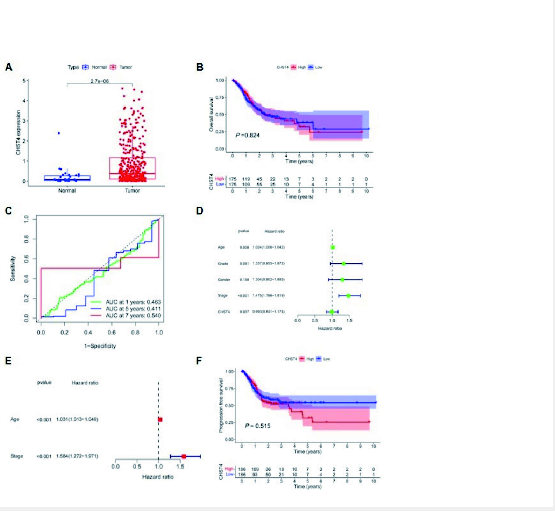

Supplement: Supplementary file 6 — Additional file 6: Figure S6. Survival curve and ROC curve to evaluate the predictive value of CHST4 expression on the prognosis of GC patients. A: box line plot of the difference in CHST4 expression in GC tissues and normal gastric mucosal tissues; B: Kaplan–Meier survival analysis curves of GC patients in the high CHST4 expression group and low CHST4 expression group; C: time-dependent ROC curves to evaluate the predictive efficacy of CHST4 on the prognosis of GC patients; D: single-factor independent prognostic analysis forest plot; E: multi-factor independent prognostic analysis forest plot; F: survival curves to evaluate the predictive efficacy of CHST4 on PFS in GC patients. [file 12967_2023_4658_MOESM6_ESM.docx]

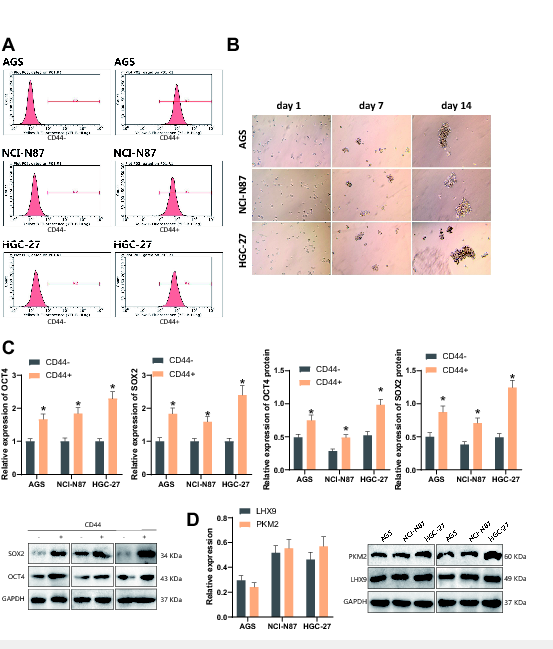

Supplement: Supplementary file 7 — Additional file 7: Figure S7. Screening and identification of GCSCs by flow cytometry, spherical assay, RT-qPCR, and Western Blot. A: flow cytometry to detect the expression of stemness marker CD44 in three GC cell lines (AGS, NCI-N87, HGC-27); B: a spheroid assay to detect spheroid formation on day 1, day 7, and day 14 in three GC cell lines (AGS, NCI-N87, HGC-27); C: RT-qPCR and Western Blot assay to detect the expression of tumor stemness markers OCT4 and SOX2 in the three GC cell lines (AGS, NCI-N87, HGC-27); D: Western Blot assay to detect the expression of LHX9 and PKM2 in the three GC cell lines (AGS, NCI-N87, HGC-27); *: P < 0.05, statistically significant for comparison between groups significance; The data comparison between the two groups was conducted using an independent samples t-test, and the cell experiments were repeated three times. [file 12967_2023_4658_MOESM7_ESM.docx]
