# Supplementary material for: Transcription factor LHX9 (LIM Homeobox 9) enhances pyruvate kinase PKM2 activity to induce glycolytic metabolic reprogramming in cancer stem cells, promoting gastric cancer progression
Source: J Transl Med. 2023 Nov 18;21:833. doi: 10.1186/s12967-023-04658-7 (PMC10657563; doi:10.1186/s12967-023-04658-7)
Supplement: Supplementary file 8 — Additional file 8: Table S1. Source information of the primary antibodies. Table S2. Lentivirus transduction sequences. Table S3. RT-qPCR primer sequences. Table S4. Source information of the primary and secondary antibodies. Table S5. Single gene prognostic analysis among six common genes. Table S6. Gene Set Enrichment Analysis (GSEA). [file 12967_2023_4658_MOESM8_ESM.docx]

**Table S1** Source information of the primary antibodies.

| Antibodies | Article No. | Company | Country | Dilution ratio |
| --- | --- | --- | --- | --- |
| LHX9 | PAB29907 | Abnova | USA | 1∶250 |
| PKM2 | 4053 | Cell Signaling Technology | USA | 1:400-1:1600 |
| CD44 | sc18849 | Santa Cruz Biotechnology | USA | 1:50-1:500 |
| GLUT-1 | ab115730 | Abcam | US | 1: 250-1: 500 |

**Table S2** Lentivirus transduction sequences.

| Group | Sequences |
| --- | --- |
| sh-NC | Nonsense sequences |
| sh-LHX9-#1 | 5’-CCTCACAAACTACCTTAACAA-3’ |
| sh-LHX9-#2 | 5’-GCAAGGAGGATTACTACAGAA-3’ |
| sh-LHX9-#3 | 5’-CCGGACCATGAAATCCTACTT-3’ |
| sh-PKM2-#1 | 5’-GTTCGGAGGTTTGATGAAATC-3’ |
| sh-PKM2-#2 | 5’-GCTGTGGCTCTAGACACTAAA-3’ |
| sh-PKM2-#3 | 5’-CGTGGATGATGGGCTTATTTC-3’ |

**Table S3** RT-qPCR primer sequences

| Genes | Sequences |
| --- | --- |
| LHX9 (human) | Forward: 5’-GGGCTGAGAAGACCTAACCG-3’ |
|  | Reverse: 5’-ACGATGCTGTTGCCGATGAA-3’ |
| GLUT1 (human) | Forward: 5’-TGTGGGCATGTGCTTCCAGTA-3’ |
|  | Reverse: 5’-CGGCCTTTAGTCTCAGGAACTTTG-3’ |
| PKM2 (human) | Forward: 5’-GACTGCCTTCATTCAGACCCA-3’ |
|  | Reverse: 5’-GGGTGGTGAATCAATGTCCAG-3’ |
| OCT4 (human) | Forward: 5'-CCTGAAGCAGAAGAGGATC-3' |
|  | Reverse: 5'-CGTTTGGCTGAATACCTT-3' |
| SOX2 (human) | Forward: 5'-ACACCAATCCCATCCACACT-3' |
|  | Reverse: 5'-GCAAACTTCCTGCAAAGCTC-3' |
| HK2 (human) | Forward: 5'-CTATGTCGAACCCTTGCCACTC-3' |
|  | Reverse: 5'-GCAAAATGGTTTAGGAAGCGG-3′ |
| LDHA (human) | Forward: 5'-TTGACCTACGTGGCTTGGAAG-3 |
|  | Reverse: 5'-GGTAACGGAATCGGGCTGAAT-3 |
| PKD1 (human) | Forward: 5'-GAAGCAGTTCCTGGACTTCG-3′ |
|  | Reverse: 5'-ACCAATTGAACGGATGGTGT-3′ |
| GAPDH (human) | Forward: 5'-GCACCGTCAAGGCTGAGAAC-3' |
|  | Reverse: 5'-TGGTGAAGACGCCAGTGGA-3′ |

**Table S4** Source information of the primary and secondary antibodies

| Antibodies | Article No. | Company | Company | Dilution ratio |
| --- | --- | --- | --- | --- |
| Mouse anti-LHX9 antibody | sc-515059 | Santa Cruz Biotechnology | USA | 1: 100-1: 1000 |
| Rabbit anti-PKM2 antibody | 4053 | Cell Signaling Technology | USA | 0.736111111 |
| Rabbit anti-OCT4 antibody | ab19857 | Abcam | USA | 1µg/ml |
| Rabbit anti-SOX2 | ab92494 | Abcam | USA | 1:1000-1:2000 |
| Rabbit anti-GLUT1 | ab115730 | Abcam | USA | 1: 100000 |
| Mouse anti-HK2 | sc-374091 | Santa Cruz Biotechnology | USA | 1: 100-1: 1000 |
| Mouse anti-LDHA | sc-133123 | Santa Cruz Biotechnology | USA | 1:100-1:1000 |
| Mouse anti-PKD1 | ab110025 | Abcam | USA | 1:500-1:2000 |
| Rabbit anti-mouse IgG | ab6728 | Abcam | USA | 1: 2000-1: 10000 |
| Goat anti-rabbit IgG | ab6721 | Abcam | USA | 3.513888889 |

**Table S5** Single gene prognostic analysis among six common genes

| Genes | Difference* | Overall survival* | ROC# | | | UniCOX* | MultiCOX* | PFS* |
| --- | --- | --- | --- | --- | --- | --- | --- | --- |
|  |  |  | 1 | 5 | 7 |  |  |  |
| MET | < 0.001 | 0.296 | 0.50 | 0.67 | 0.76 | 0.17 | > 0.05 | 0.358 |
| TKTL1 | < 0.001 | 0.134 | 0.47 | 0.45 | 0.44 | 0.34 | > 0.05 | 0.911 |
| CLDN9 | < 0.001 | 0.006 | 0.53 | 0.65 | 0.71 | 0.00 | < 0.05 | 0.054 |
| MIOX | < 0.001 | 0.964 | 0.50 | 0.60 | 0.77 | 0.85 | > 0.05 | 0.056 |
| LHX9 | < 0.001 | 0.031 | 0.55 | 0.66 | 0.77 | 0.13 | > 0.05 | 0.022 |
| CHST4 | < 0.001 | 0.740 | 0.46 | 0.41 | 0.54 | 0.94 | > 0.05 | 0.239 |

Note: Differential analysis, overall survival, ROC for different survival years (1, 5, and 7 years) of GC patients, UniCOX, UniCOX, and progress free interval were performed on six candidate genes. * indicates *p* value; # indicates area under ROC curve.

**Table S6** Gene Set Enrichment Analysis (GSEA)

| **Pathway Name** | **Enrichment Score (ES)** | **Normalized Enrichment Score (NES)** | **FDR** |
| --- | --- | --- | --- |
| HALLMARK GLYCOLYSIS | 0.5189426 | 1.5654967 | 0.08598131 |
| REACTOME GLYCOLYSIS | 0.7086662 | 1.8818998 | 0.0 |
| BIOCARTA GLYCOLYSIS PATHWAY | 0.5357753 | 0.8653614 | 0.68191266 |
